# The value of MRI examination on bilateral hands including proximal interphalangeal joints for disease assessment in patients with early rheumatoid arthritis: a cross-sectional cohort study

**DOI:** 10.1186/s13075-019-2061-1

**Published:** 2019-12-11

**Authors:** Ying-Qian Mo, Ze-Hong Yang, Jun-Wei Wang, Qian-Hua Li, Xin-Yun Du, T. W. Huizinga, X. M. E. Matthijssen, Guang-Zi Shi, Jun Shen, Lie Dai

**Affiliations:** 1 Department of Rheumatology, Sun Yat-Sen Memorial Hospital, Sun Yat-Sen University, Guangzhou, People’s Republic of China; 2 Department of Radiology, Sun Yat-Sen Memorial Hospital, Sun Yat-Sen University, Guangzhou, People’s Republic of China; 3 Department of Rheumatology, Leiden University Medical Center, Leiden, Netherlands

**Keywords:** Arthritis, Rheumatoid arthritis, Magnetic resonance imaging, Rheumatoid arthritis magnetic resonance imaging score (RAMRIS), Tenosynovitis

## Abstract

**Background:**

Bilateral hands including proximal interphalangeal joints (PIPJs) are recommended on physical, X-ray radiographic, or ultrasonographic examination by clinical guidelines of rheumatoid arthritis (RA), but MRI still tends to examine unilateral wrists and/or MCPJs. We aimed to demonstrate the advantages of MRI examination on bilateral hands including PIPJs for disease assessment in early RA patients.

**Methods:**

Active early RA patients received 3.0T whole-body MRI examination with contrast-enhanced imaging on bilateral wrists, MCPJs, and PIPJs. MRI features were scored referring to the updated RAMRIS. Clinical assessments were conducted on the day of MRI examination.

**Results:**

The mean time of MRI examination was 24 ± 3 min. MRI bone erosion in MCPJs would be missed-diagnosed in 23% of patients if non-dominant MCPJs were scanned unilaterally, while osteitis in MCPJs would be missed-diagnosed in 16% of patients if dominant MCPJs were scanned unilaterally. MRI synovitis severity was also asymmetric: 21% of patients showing severe synovitis unilaterally in non-dominant MCPJs/PIPJs and other 20% showing severe synovitis unilaterally in dominant MCPJs/PIPJs. Among these early RA patients, MRI tenosynovitis occurred the most frequently in wrist extensor compartment I, while MRI examination on bilateral hands demonstrated no overuse influence present. However, overuse should be considered in dominant PIPJ2, PIPJ4, and IPJ of thumb of which MRI tenosynovitis prevalence was respectively 18%, 17%, or 16% higher than the non-dominant counterparts. Early MRI abnormality of nervus medianus secondary to severe tenosynovitis occurred either in dominant or non-dominant wrists; MRI of unilateral hands would take a risk of missed-diagnosis. Common MRI findings in PIPJs were synovitis and tenosynovitis, respectively in 87% and 69% of patients. MRI tenosynovitis prevalence in IPJ of thumb or PIPJ5 was much higher than the continued wrist flexor compartments. MRI synovitis or tenosynovitis in PIPJs independently increased more than twice probability of joint tenderness (OR = 2.09 or 2.83, both *p* < 0.001).

**Conclusions:**

In consideration of asymmetric MRI features in early RA, potential overuse influence for certain tenosynovitis in dominant hands, and high prevalence of MRI findings in PIPJs, MRI examination on bilateral hands including PIPJs is deserved for disease assessment in early RA patients.

## Background

Rheumatoid arthritis (RA) is characterized by autoimmune inflammation involving synovium, articular tendons, and subchondral bone, which affects metacarpophalangeal joints (MCPJs), proximal interphalangeal joints (PIPJs), and wrists the most commonly at early stage. Physical examination on bilateral hands contributes to indispensable parameters (e.g., tender or swollen joint count) in composite RA disease activity measures [1]. Regarding differential diagnosis between RA and other polyarthritis, X-ray radiographic and ultrasonographic examinations give preference to bilateral hands instead of unilateral hands. Various versions of X-ray scoring systems (e.g., Larsen scores or Sharp scores [2, 3]) in assessment of bone erosion or joint space narrowing in RA evaluate not only bilateral MCPJs and wrists, but also PIPJs. Superior to X-ray radiography, magnetic resonance imaging (MRI) can visualize synovitis and tenosynovitis and is the only imaging procedure to evaluate osteitis which is a strong independent predictor of subsequent radiographic progression in early RA [4]. However, MRI still tends to examine unilateral wrists and/or MCPJs for RA patients. One of the initial explanations was that bilateral MCPJs and wrists had to be scanned separately in two or four fields of view if using extremity MRI, which doubled or quadrupled the scanning time. Owing to the recent development of whole-body MRI with multichannel synergic coils and higher signal to noise ratio than extremity MRI, all RA-relevant MRI features are demonstrated more distinctly than before, while bilateral hands including PIPJs can be scanned in one field of view (FOV) to shorten the scanning time [5–8]. Compared to the original RA-MRI Scoring (RAMRIS) which was generated from databases only containing images of dominant wrists and/or MCPJs [9, 10], there is a new change in the 2016 updated RAMRIS that dominant hand has not been stressed and recommended [11]. Nevertheless, whether MRI examination should be performed on bilateral hands in RA patients has not been mentioned yet, and PIPJs are still not contained in the updated RAMRIS.

MRI examination on bilateral hands of RA patients is reported scarcely till now. Two studies which lack T2-weighted and contrast-enhanced images only evaluated bone erosion [12, 13]. Navalho et al. used 3.0T MRI examination on bilateral hands and identified a higher prevalence of synovitis or tenosynovitis detected by MRI than ultrasonography in an early polyarthritis cohort [5]. They also concluded that early RA was an asymmetric disease according to MRI tenosynovitis in bilateral hands [6] which was assessed with an empirical score for tenosynovitis described by Haavardsholm et al. [14]. Our prior study revealed the dominant or clinically more severe hands could not represent the contralateral hands to evaluate RAMRIS in a cohort mainly made up of established RA patients rather than early RA patients, while MRI tenosynovitis and PIPJs were not studied [7]. As an extension and development, here we put forward an examination regimen of high-field (3.0 T) whole-body MRI to scan bilateral wrists, MCPJs, and PIPJs in one field of view in a cohort of early RA patients and adopted the official tenosynovitis score recommended by the 2016 updated RAMRIS [11, 15]. This is the first study describing the difference of MRI features between dominant and non-dominant hands, as well as MRI features in PIPJs and their concordance with physical examination in early RA patients, which revealed MRI features including vital findings (e.g., abnormality in nervus medianus) are not always symmetric, overuse may influence certain tenosynovitis in dominant hands, and MRI findings in PIPJs were prevalent; thus, MRI examination on bilateral hands including PIPJs is deserved for disease assessment of early RA.

## Patients and methods

### Patients

Early RA patients who fulfilled 2010 American College of Rheumatology (ACR)/European League Against Rheumatism (EULAR) classification criteria [16], with active disease defined as disease activity score on 28-joint counts with C-reactive protein (DAS28-CRP) ≥ 2.6 [17] and disease duration no more than 1 year, were recruited during April 2014 to March 2018 at Department of Rheumatology, Sun Yat-Sen Memorial Hospital, Sun Yat-Sen University. The recruited patients who were willing to receive MRI examination on bilateral hands signed a written informed consent which was approved by the Medical Ethics Committee of Sun Yat-sen Memorial Hospital (SYSEC-2009-06 and SYSEC-KY-KS-012). Patients were excluded if they had contraindications to contrast agent or MRI images were unqualified. This study was conducted in compliance with the Helsinki Declaration.

### Clinical and X-ray radiographic assessment

On the day of MRI examination, demographic and clinical data were collected as we described previously [18, 19] including gender, age, disease duration, 28-joint tender and swollen joint count (28TJC and 28SJC), patient and provider global assessment of disease activity (PtGA and PrGA, range 0–10), CRP (mg/L, 0–5 mg/L), erythrocyte sedimentation rate [ESR, mm/h, 0–20 mm/h (female), 0–15 mm/h (male)], rheumatoid factor (RF, mg/L, 0–20 mg/L), anti-citrullinated peptide antibody (ACPA, IU/ml, 0–18 IU/ml), and prior therapy. Disease activity was assessed with DAS28-CRP, clinical disease activity index (CDAI), and simplified disease activity index (SDAI). Functional limitation was defined as Stanford Health Assessment Questionnaire-Disability Index (HAQ-DI)≥1 [20, 21]. Conventional radiographs of bilateral hands (anteroposterior view) were used to assess the Sharp/van der Heijde modified total Sharp score (mTSS), joint space narrowing subscore (JSN), and erosion subscore as we described previously [22, 23], while bone erosion in X-ray was defined as a discrete interruption of the cortical surface.

### MRI imaging and assessment

Bilateral wrists, MCPJs, and PIPJs of each patient were scanned by 3.0T whole-body MRI in one field of view of a 25-cm eight-channel sense head coil (Achieva; Philips Medical Systems, the Netherland). Each patient was imaged in a prone position with pronation of bilateral hands side by side over the head with the help of three or more 2-kg sandbags. The MRI sequences had been described in our prior study [7], which comprised coronal turbo spin echo fat-suppressed T2-weighted imaging [time of repetition (TR), 2718.2 ms; time of echo (TE), 30 ms; slice thickness/gap, 2.5/0 mm; FOV, 128 × 128; matrix, 312 × 312], coronal spin echo T1-weighted imaging (TR, 500 ms; TE, 15 ms; slice thickness/gap, 2.5/0 mm; FOV, 128 × 128; matrix, 356 × 275), axial turbo spin echo fat-suppressed T2-weighted imaging (TR, 3443.7 ms; TE, 30 ms; slice thickness/gap, 5/2 mm; FOV, 128 × 128; matrix, 312 × 310). Contrast-enhanced imaging was initiated immediately after intravenous injection of 0.2 mmol/kg gadolinium (Gd)-DTPA (Magnevist, Bayer Pharma AG, Germany), with imaging sequences of axial spin echo fat-suppressed T1-weighted imaging (TR, 500 ms; TE, 15 ms; slice thickness/gap, 5/2 mm; FOV, 128 × 128; matrix, 190 × 312) and coronal spin echo fat-suppressed T1-weighted imaging (TR, 500 ms; TE, 15 ms; slice thickness/gap, 2.5/0 mm; FOV, 128 × 128; matrix, 275 × 356).

All MRI images were scored by two experienced radiologists (Yang ZH and Shi GZ, with previous experience on musculoskeletal MRI [7]) who were blinded to clinical data and read twice on identical screens over 2 days. MRI tenosynovitis, synovitis, osteitis, and bone erosion were scored referring to the 2016 updated RAMRIS [11]. Tenosynovitis score in ten tendon compartments at the wrist level and digit flexor tendons at the level of MCPJs or PIPJs was graded according to the maximum width of peritendinous effusion and/or postcontrast enhancement of the tendon sheath visualized on axial sequences over ≥ 3 consecutive slices, as follows: 0, absent; 1, < 1.5 mm; 2, ≥ 1.5 mm but < 3 mm; 3, ≥ 3 mm. The presence of MRI tenosynovitis was defined as score ≥ 1. The mean intra-class correlation coefficient (ICC) of intra-observer agreement was 0.9 which indicated very good agreement, and the mean ICC of inter-observer agreement was 0.7 which indicated good agreement [24].

### Statistical analysis

Statistical analyses were performed with SPSS for Windows 20.0 statistical software (SPSS Inc., Chicago, IL, USA). Data were presented as frequencies and percentages for categorical variables and mean with standard deviation (SD) or median with interquartile range (*IQR*) for continuous variables according to distributions. Spearman rank correlation analysis with significant tests on correlation indexes (*r*) was used to identify whether a correlation between two indicators was significant. Mann-Whitney rank-sum test was used for comparison between two independence groups. Chi-square test was used for categorical variables among groups. Univariate logistic regression analyses were used to identify the contribution of tenosynovitis on joint tenderness or swelling, with continuous MRI tenosynovitis score per joint as independent variables. To confirm the independent risk of tenosynovitis on joint tenderness or swelling, generalized estimating equations (GEE) with multivariate logistic regression analyses were used, with continuous MRI tenosynovitis, synovitis, and osteitis score per joint as independent variables. All significance tests were two-tailed and were conducted at the 5% significance level, unless otherwise specified.

## Results

### Baseline characteristics of early RA patients

A total of 83 early RA patients received MRI examination of bilateral hands. Eight of them were excluded due to incomplete images of MCPJs and/or PIPJs. Finally, 75 patients were qualified for statistical analyses (Table 1), including 68 (91%) hospitalized in-patients and 7 (9%) outpatients. There were 51%, 44%, and 5% of patients respectively in high, moderate, and low disease activity according to DAS28-CRP. Forty-four patients (59%) were treatment-naïve who had never taken any disease-modifying anti-rheumatic drugs (DMARDs) or glucocorticoids before recruitment.

**Table 1:** Demographic and clinical characteristics of 75 patients with early rheumatoid arthritis

Characteristics	Early RA *N* = 75
Female, *n* (%)	53 (71)
Age, years, median (*IQR*)	49 (38–59)
Disease duration, months, median (*IQR*)	7 (3–12)
Core disease activity indexes	
28TJC, median (*IQR*)	8 (5–13)
28SJC, median (*IQR*)	6 (3–9)
PtGA, median (*IQR*)	5.5 (4–8)
PrGA, median (*IQR*)	5 (4–7)
ESR, mm/h, median (*IQR*)	67 (38–81)
CRP, mg/L, median (*IQR*)	39 (13–63)
RF positive rate, *n* (%)	53 (71)
ACPA positive rate, *n* (%)	56 (75)
DAS28-CRP, median (*IQR*)	5.1 (4.2–6.1)
SDAI, median (*IQR*)	28 (20–41)
CDAI, median (*IQR*)	25 (17–34)
HAQ-DI score, median (*IQR*)	1.0 (0.3–1.8)
Functional limitation^△^, *n* (%)	38 (51)
Radiographic assessment	
mTSS, median (*IQR*)	8 (3–25)
JSN subscore, median (*IQR*)	1 (0–4)
Erosion subscore, median (*IQR*)	6 (2–22)
Bone erosions, *n* (%)	64 (85)
Prior therapy during 3 months before recruitment	
Treatment-naïve^△△^, *n* (%)	44 (59%)
Glucocorticoids, *n* (%)	19 (25)
Methotrexate, *n* (%)	18 (24)
Leflunomide, *n* (%)	8 (11)
Salazosulfadimidine, *n* (%)	4 (5)
hydroxychloroquine, *n* (%)	6 (8)
Biological DMARDs, *n* (%)	3 (4)
Chinese patent medicine, *n* (%)	12 (16)

*IQR* Interquartile range, *28TJC* 28-joint tender joint count, *28SJC* 28-joint swollen joint count, *PtGA* patient global assessment of disease activity, *PrGA* provider global assessment of disease activity, *ESR* erythrocyte sedimentation rate, *CRP* C-reactive protein, *RF* rheumatoid factor, *ACPA* anti-cyclic citrullinated peptide antibody, *DAS28* disease activity score in 28 joints, *SDAI* simplified disease activity index, *CDAI* clinical disease activity index, *HAQ-DI* Health Assessment Questionnaire-Disability Index, *mTSS* modified total Sharp score, *JSN* joint space narrowing, *DMARDs* disease-modifying antirheumatic drugs

^△^Functional limitation was defined as HAQ-DI score ≥ 1 [17]

^△△^Patients of treatment-naïve were defined as who had never taken any DMARDs or glucocorticoids before recruitment

### RAMRIS in bilateral hands correlate with indexes of disease activity or radiographic assessment

The mean time of entire MRI examination on bilateral hands containing patient positioning and contrast agent injection was 24 ± 3 min. RAMRIS of synovitis, tenosynovitis, osteitis, and bone erosion in bilateral hands of 75 early RA patients were 23 (IQR, 13~32), 15 (IQR, 4~24), 21 (IQR, 3~40), and 27 (*IQR*, 10~37), respectively. RAMRIS of synovitis was significantly correlated with 28TJC, 28SJC, ESR, CRP, DAS28-CRP, SDAI, and CDAI (*r* = 0.262~0.400, all *p* < 0.05, Additional file 1: Table S1); RAMRIS of tenosynovitis was slightly but significantly correlated with 28TJC, CRP, DAS28-CRP, and SDAI (*r* = 0.269~0.282, all *p* < 0.05); and osteitis score was only slightly correlated with CRP (*r* = 0.242, *p* < 0.05). Bone erosions in bilateral hands were detected in 64 patients (85%) by X-ray, but in all patients (100%) by MRI. RAMRIS of osteitis or bone erosion in bilateral hands was moderately and positively correlated with mTSS, erosion subscore, and JSN subscore (*r* = 0.416~0.522, all *p* < 0.01, Additional file 1: Table S1). Thirty-eight patients (51%) with functional limitation had significantly higher RAMRIS of synovitis in bilateral hands than those without functional limitation [27 (IQR, 18~34) vs. 17 (IQR, 9~30), *p* < 0.05].

### The advantages of MRI examination on bilateral hands for disease assessment of early RA

MRI examination on bilateral hands showed MRI features in early RA patients were asymmetric to various extent in different joint areas, leading to missed-diagnosis if only dominant or non-dominant hands were scanned by MRI (Table 2). MRI bone erosion in MCPJs would be missed-diagnosed in 17 patients (23%) based on MRI of non-dominant hands, while osteitis in MCPJs would be missed-diagnosed in 12 patients (16%) based on MRI of dominant hands. Likewise, MRI osteitis in PIPJs or synovitis in wrists would be missed-diagnosed in 8 patients (11%) based on MRI of non-dominant hands. MRI synovitis severity was also asymmetric. Sixteen patients (21%) whose synovitis score was less than 2 per joint (mild synovitis) among dominant MCPJs and PIPJs showed severe synovitis (synovitis score ≥ 2) in at least one joint among non-dominant MCPJs and PIPJs. Similarly, other 15 patients (20%) showed mild synovitis unilaterally in non-dominant MCPJs and PIPJs.

**Table 2:** Prevalence of MRI findings and missed-diagnosis rate in different joint areas based on putative MRI of dominant or non-dominant hands compared to MRI of bilateral hands in 75 active early RA patients

	MRI of bilateral hands	Putative MRI of dominant hands^☆^		Putative MRI of non-dominant hands^☆^	
	MRI findings^△^	MRI findings^△^	Missed-diagnosis rate^▲^	MRI findings^△^	Missed-diagnosis rate^▲^
PIPJs					
Tenosynovitis	52 (69%)	50 (67%)	2 (3%)	45 (60%)	7 (9%)
Synovitis	65 (87%)	59 (79%)	6 (8%)	58 (77%)	7 (9%)
Osteitis	23 (31%)	18 (24%)	5 (7%)	15 (20%)	8 (11%)
Bone erosion	22 (29%)	17 (23%)	5 (7%)	17 (23%)	5 (7%)
MCPJs					
Tenosynovitis	55 (73%)	52 (69%)	3 (4%)	46 (61%)	9 (12%)
Synovitis	64 (85%)	64 (85%)	0	61 (81%)	3 (4%)
Osteitis	46 (61%)	34 (45%)	12 (16%)	39 (52%)	7 (9%)
Bone erosion	50 (67%)	42 (56%)	8 (11%)	33 (44%)	17 (23%)
Wrists					
Tenosynovitis	56 (75%)	54 (72%)	2 (3%)	51 (68%)	5 (7%)
Synovitis	72 (96%)	72 (96%)	0	64 (85%)	8 (11%)
Osteitis	59 (79%)	52 (69%)	7 (9%)	52 (69%)	7 (9%)
Bone erosion	74 (99%)	72 (96%)	2 (3%)	74 (99%)	0

*MCPJs* metacarpophalangeal joints, *PIPJs* proximal interphalangeal joints

^☆^Putative MRI of dominant or non-dominant hands means analyses on dominant or non-dominant hands based on MRI of bilateral hands, rather than performing separate MRI of dominant or non-dominant hands which increases physical and financial burdens of patients

^△^*n* = the number of patients with MRI findings in a certain joint region (e.g., PIPJs, MCPJs or wrists)

^▲^*n* = the number of patients whose diagnosis would be missed if dominant or non-dominant hands were evaluated unilaterally

**Fig. 1:**
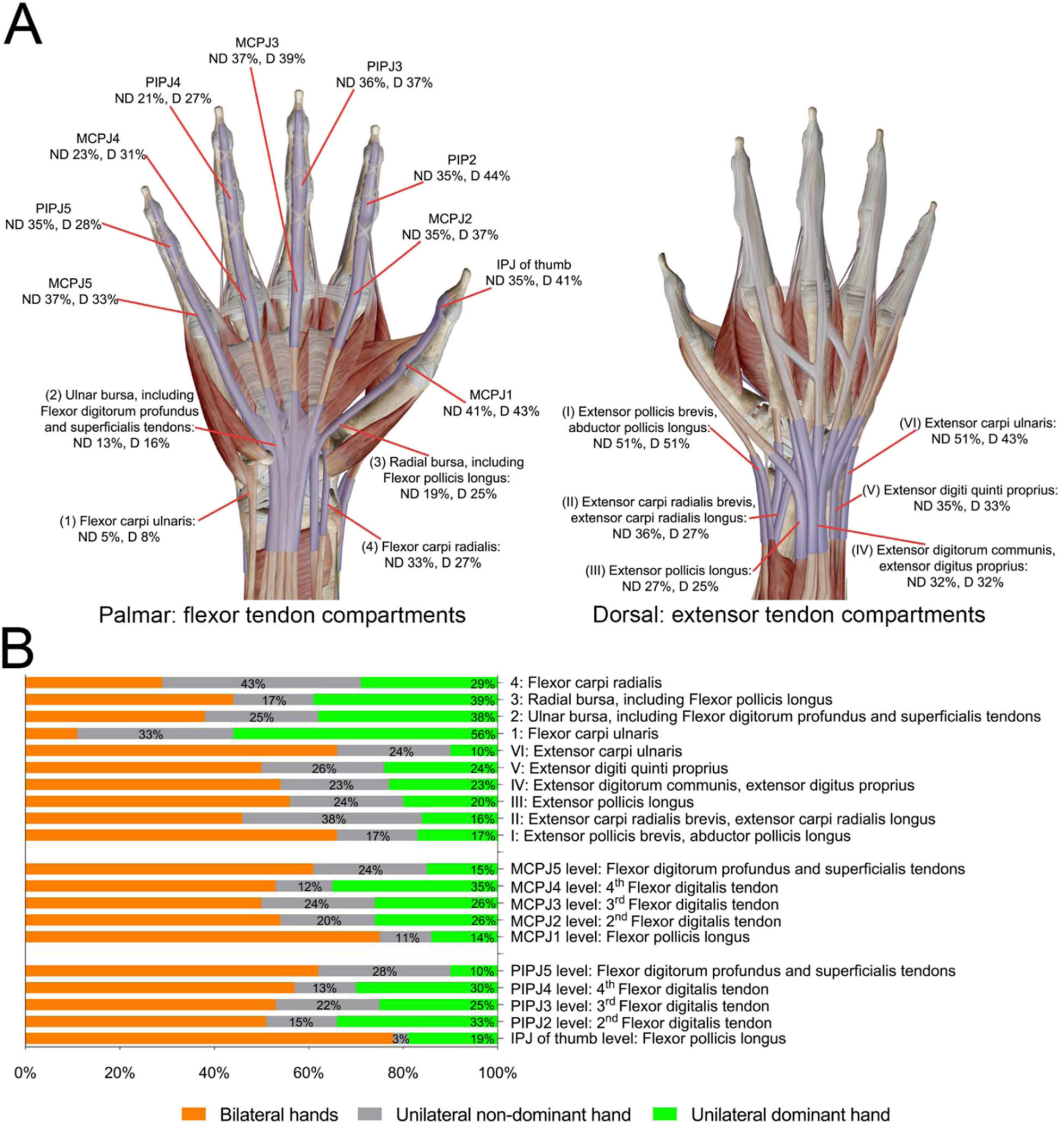
The prevalence and involvement pattern of MRI tenosynovitis in bilateral hands. **a** The prevalence of MRI tenosynovitis per tendon compartment or per joint of 75 dominant or nondominant hands. **b** The involvement pattern of MRI tenosynovitis in ten wrist tendon compartments and digit flexor tenosynovitis in MCPJs or PIPJs. ND, non-dominant; D, dominant; IPJ, interphalangeal joint; PIPJ, proximal interphalangeal joint; MCPJ, metacarpophalangeal joint

Among 75 early RA patients, MRI tenosynovitis occurred the most frequently in the wrist extensor compartment I where De Quervain tenosynovitis can occur due to activities involving repeated radioulnar deviation (e.g., hammering or lifting heavy things). Upon comparison of MRI tenosynovitis prevalence per tendon compartment between dominant and non-dominant counterparts, we preliminary assessed overuse influence for tenosynovitis in dominant hands (Fig. 1). Tenosynovitis prevalence in either dominant or non-dominant wrist extensor compartment I was equal, so overuse seemed to play little role on this tendon compartment in early RA patients. Among 39 patients who had MRI tenosynovitis in PIPJ2, tenosynovitis prevalence in dominant PIPJ2 was 18% higher than that in non-dominant PIPJ2 (Fig. 1b, “percentages in green” subtracted “percentages in grey”). Likewise, MRI tenosynovitis prevalence in dominant PIPJ4 or interphalangeal joint (IPJ) of thumb was respectively 17% or 16% higher than that in their non-dominant counterparts. It implied MRI of dominant hands may have bias to assess tenosynovitis in PIPJ2, PIPJ4, and IPJ of thumb. Additionally, over 50% of patients who had MRI tenosynovitis in wrist flexor compartments showed unilateral tenosynovitis (Fig. 1b, “percentages in green” added “percentages in gray”).

**Fig. 2:**
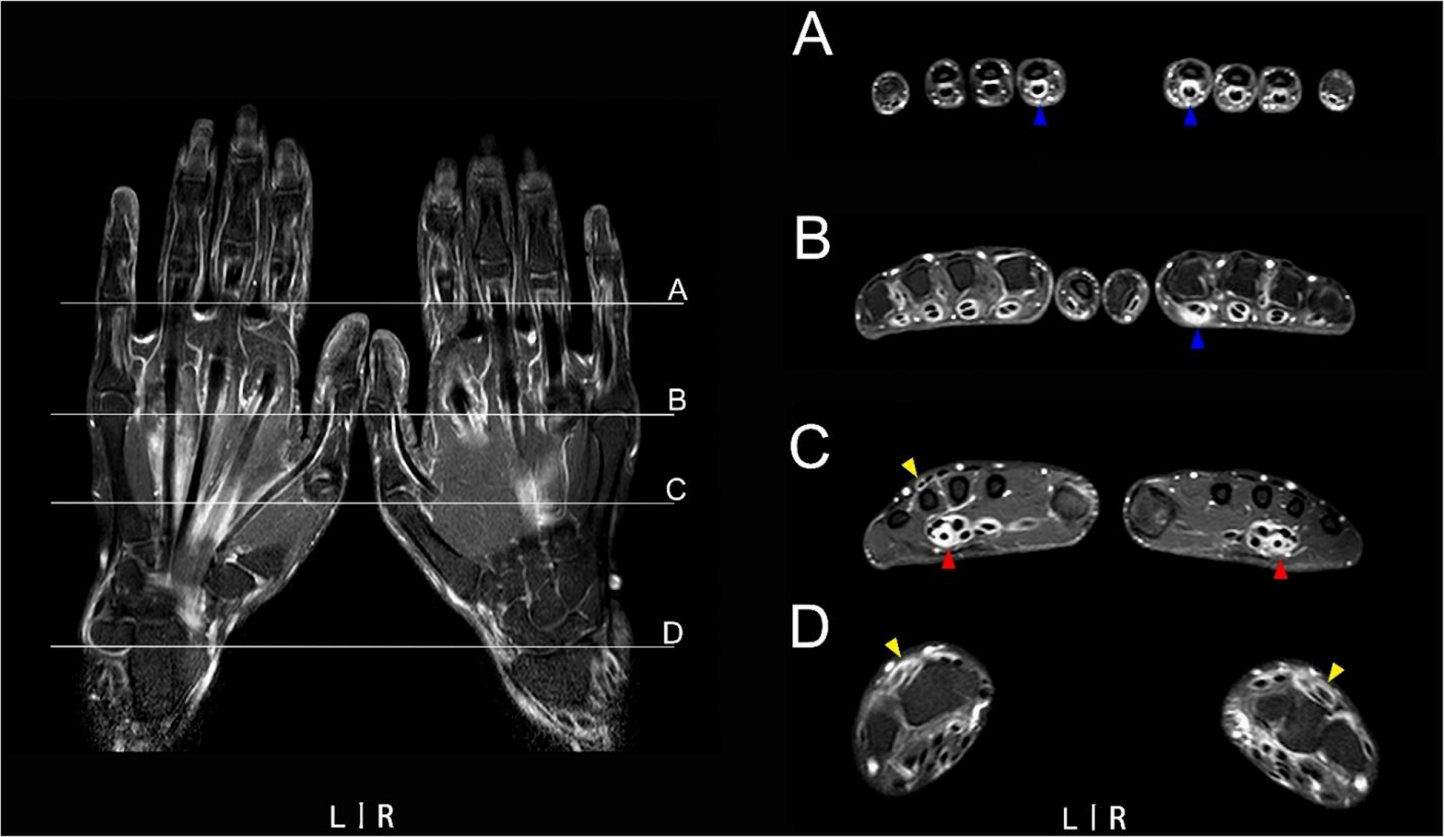
A typical MRI image of tenosynovitis. The patient was a 29-year-old female with 11 months of RA disease duration and DAS28-CRP of 4.52, and she never took any disease-modifying anti-rheumatic drugs or glucocorticoid. Left, coronal spin echo fat-suppressed T1-weighted imaging; right (A–D), axial spin echo fat-suppressed T1-weighted imaging. (A) Proximal interphalangeal joints 2~5; (B) interphalangeal joint of thumb and metacarpophalangeal joints 2~5; (C) metacarpophalangeal joint 1 and the distal part of wrist; and (D) the proximal part of wrist. Blue arrows, flexor tenosynovitis of digit in PIPJ or MCPJ; red arrows, tenosynovitis in bilateral wrist flexor compartments 2; yellow arrows, tenosynovitis in bilateral wrist extensor compartments IV

Wrist flexor compartments 2 (red arrows in Fig. 2c~d) and flexor compartments 3 received additional attention since they pass through the carpal tunnel in the wrist. MRI of bilateral hands demonstrated severe tenosynovitis (tenosynovitis score ≥ 2) simultaneously in wrist flexor compartments 2 and 3 was detected in three dominant wrists and two non-dominant wrists. Four of the above five wrists (80%) showed marked peritendinous effusion inside carpal tunnel along with irritation of nervus medianus under MRI. Since the early abnormality of carpal tunnel syndrome occurred either in dominant or non-dominant wrists, MRI of unilateral hands would take a risk of missed diagnosis.

### MRI features of PIPJs in early RA patients

**Fig. 3:**
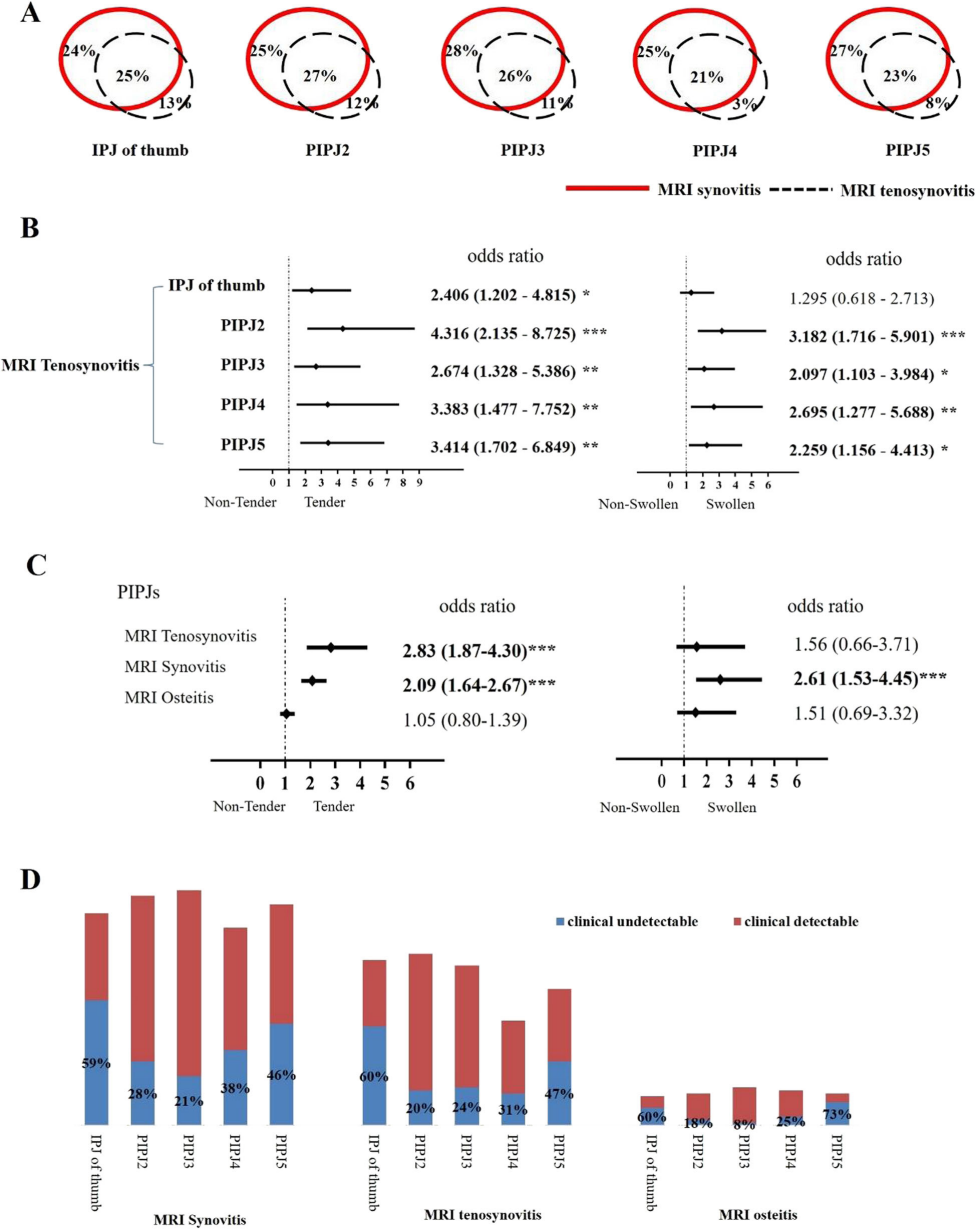
The relationship between MRI inflammation and tenderness or swelling detected by physical examination in PIPJs of 75 early RA patients. **a** Venn diagrams showed relations between MRI synovitis and tenosynovitis in each type of PIPJs. **b** Univariate logistics regression analyses showed the contribution of MRI tenosynovitis to joint tenderness or swelling in each type of PIPJs. **c** Multivariate logistic regression in generalized estimating equations showed the contribution of each MRI inflammation to joint tenderness or swelling in PIPJs. **d** The rate of subclinical MRI inflammation which was clinical undetectable in each type of PIPJs

There were few data on MRI features of PIPJs in early RA patients, so we included PIPJs in MRI examination of bilateral hands in this study. The common MRI findings in PIPJs were synovitis and tenosynovitis which occurred respectively in 87% and 69% of 75 early RA patients, while osteitis or bone erosion in PIPJs occurred only in 27% or 29% of patients. Among all 750 PIPJs, 50%, 34%, 7.6%, and 10% of them respectively showed MRI synovitis, tenosynovitis, osteitis, and bone erosion. Although synovitis was present in the articular capsule of PIPJs and tenosynovitis was present in digit flexor tendon at the palm of PIPJs, synovitis and tenosynovitis occurred together in 25% of IPJs of thumb, 27% of PIPJ2, 26% of PIPJ3, 21% of PIPJ4, and 23% of PIPJ5 (Fig. 3a). Severe synovitis (synovitis score ≥ 2) was detected in 17% of PIPJs, while only 5% of PIPJs showed severe tenosynovitis (tenosynovitis score ≥ 2).

Digit flexor tendons of PIPJ2~PIPJ4 are isolated and respectively have a gap apart from the tenosynovium in the carpal tunnel. Digit flexor tendon in IPJ of thumb derives from the 3rd wrist flexor compartment (flexor pollicis longus). The tenosynovitis prevalence in either dominant or non-dominant IPJ of thumb was 16% higher than the continued 3rd wrist flexor compartment (Fig. 1a). Likewise, digit flexor tendon in PIPJ5 derives from the 2nd wrist flexor compartment (flexor digitorum profundus and superficialis tendons). The tenosynovitis prevalence in dominant and non-dominant PIPJ5 was 12% and 22% respectively higher than the continued 2nd wrist flexor compartment (Fig. 1a).

### Concordance between MRI inflammation and physical examination in PIPJs

PIPJs were divided into three groups: synovitis together with tenosynovitis, synovitis alone, and no synovitis. The prevalence of tenderness or swelling was compared among these three groups per joint type (e.g., all PIPJ2), and *p* < 0.0167 was considered as significantly different between any two groups. The prevalence of tenderness among IPJs of thumb or PIPJ2 with synovitis plus tenosynovitis was significantly higher than the corresponding joints with synovitis alone (IPJs of thumb, 46% v.s. 19%; PIPJ2, 83% v.s. 53%, all *p* < 0.0167). Univariate logistics regression analyses showed MRI tenosynovitis in any type of PIPJs significantly contributed to tenderness (Fig. 3b). GEE with multivariate logistic regression which combined various types of PIPJs and adjusted confounding impact of different MRI inflammation confirmed that not only MRI synovitis but also tenosynovitis independently increased more than twice probability of joint tenderness (all *p* < 0.001, Fig. 3c).

Among 377 PIPJs with MRI synovitis, 38% of them were clinically undetectable, neither swollen nor tender on physical examination. Likewise, 36% of 254 PIPJs with MRI tenosynovitis and 35% of 57 PIPJs with MRI osteitis were clinically undetectable. The rates of subclinical MRI inflammation in IPJ of thumb or PIPJ5 were higher than other PIPJs (Fig. 3d). Even for PIPJs with severe MRI inflammation (score ≥ 2), there were still 27% of 127 PIPJs with severe synovitis, 18% of 40 PIPJs with severe tenosynovitis, and 28% of 32 PIPJs with severe osteitis were clinically undetectable.

Among 424 PIPJs which were neither swollen nor tender on physical examination, there were still 33%, 21%, and 5% of them respectively showing MRI synovitis, tenosynovitis, and osteitis, and 8%, 2%, and 2% of them showed severe MRI inflammation (score ≥ 2).

## Discussion

This is the first report which revealed the value of MRI examination on bilateral hands including PIPJs for disease assessment in a cohort of 75 early RA patients. It was unexpected that MRI features in early RA patients were asymmetric to various extent in different joint areas, leading to missed-diagnosis of MRI findings if either dominant or non-dominant hands were scanned instead of bilateral hands, especially in MCPJs. MRI bone erosion in MCPJs would be missed-diagnosed in 23% of patients based on MRI of non-dominant hands, while osteitis in MCPJs would be missed-diagnosed in 16% of patients based on MRI of dominant hands. MCPJs’ involvement weighted heavily in arthralgia suspicious for progression to RA [25] and was contained in various classification criteria of RA diagnosis. MCPJs therefore should be assessed bilaterally by MRI in patients suspicious to RA. Synovitis has a high value in diagnosis and disease activity assessment of RA. MRI of bilateral hands in our study revealed synovitis severity in early RA patients was not always symmetric. There were 21% of patients showing severe MRI synovitis unilaterally in non-dominant MCPJs/PIPJs, and other 20% of patients showed severe synovitis unilaterally in dominant MCPJs/PIPJs, that meant MRI examination on either unilateral hands may cause misevaluation on synovitis severity in one fifth of early RA patients.

Hand tenosynovitis in patients with early arthritis or early RA was significantly associated with morning stiffness, impairment of hand function, and hand dexterity [26–29]. Given the advanced performance of MRI on visualizing tendons and inflammation, all kinds of quantification or scoring systems for MRI tenosynovitis in hands were shaped constantly [14, 30–34]. The 2016 updated RAMRIS first launched a standardized score system for MRI tenosynovitis in hands and recommended tenosynovitis as well as synovitis, osteitis, and bone erosion are crucial MRI features in management of RA [11]. The cut-off value between mild and severe tenosynovitis was 2 mm of peritendinous effusion width in previous empirical score system [14], while the updated RAMRIS decreased it to 1.5 mm [11]. Here we first reported the width of peritendinous effusion just over 1.5 mm but simultaneously in wrist flexor compartments 2 and 3 could cause early MRI abnormality in nervus medianus, especially when the tenosynovitis was partially close to nervus medianus or extend to overall tendons inside the carpal tunnel. Systematic review reported that subclinical carpal tunnel syndrome had a pooled prevalence of 14% (30/215) in RA patients [35], so rheumatologists and radiologists should be alert by the abovementioned severe tenosynovitis, and MRI of bilateral hands is deserved to detect early signs of carpal tunnel syndrome which can occur in either one hand.

It is well known that overuse may cause tenosynovitis to an extent, and one of canonical examples is De Quervain tenosynovitis in wrist extensor compartment I [36]. Nieuwenhuis et al. reported higher prevalence of tenosynovitis in wrist extensor compartment I in early polyarthritis patients with progression to RA than those without any progression [37], but they could not evaluate overuse influence based on MRI of dominant hands. Here we found MRI tenosynovitis in wrist extensor compartment I occurred the most frequently among tendon compartments in hands of early RA patients, while overuse influence turned out to be absent owing to equal prevalence of MRI tenosynovitis in dominant and non-dominant wrist extensor compartment I. We also demonstrated MRI tenosynovitis prevalence in dominant PIPJ2, PIPJ4, and IPJ of thumb was respectively 18%, 17%, or 16% higher than their non-dominant counterparts, so MRI of dominant hands was not optimal to assess tenosynovitis in these joints. Recently, to avoid potential overuse influence for tenosynovitis in dominant hands, some studies selected non-dominant hands unilaterally for ultrasonographic or MRI examinations [38, 39]. However, MRI of non-dominant hands is still one-sided compared to MRI of bilateral hands (Table 2).

Although bilateral wrists, MCPJs, and PIPJs are contained in standard examination regimens of X-ray radiography in management of RA [2, 3], dominant wrists and/or MCPJs are still preferential on MRI examination in patients with early polyarthritis or RA [26, 37, 40–43]. This study put forward an examination regimen using high-field (3.0 T) whole-body MRI with an eight-channel sense head coil, not only containing bilateral wrists, MCPJs, and PIPJs in one field of view but also speeding up imaging. The entire MRI examination of bilateral hands containing patient positioning and contrast agent injection cost less than half an hour, facilitating it into practice. Eight patients in preliminary stage of this study were excluded due to incomplete images of MCPJs and/or PIPJs. The reasons were that joints (especially PIPJs) could not keep straight during scanning, or artifacts in the joints near the edge of coil. MRI images kept qualified after two measures were strictly implemented. First, bilateral hands must be placed in the center of the coil to assure wrists, MCPJs, and PIPJs be exhibited in both coronal and axial imaging. Second, in order to keep hand joints motionless and straight, we used at least three sandbags: one beneath bilateral hands and two on both forearms.

PIPJs are usually the earliest involved region in RA patients. Digit flexor tenosynovitis (containing PIPJs) detected by ultrasonography was reported to be of independent predictive value for RA development in patients with early arthritis [44] and was also an independent risk factor of flare in RA patients with clinical remission [45]. However, PIPJs were scarcely evaluated by MRI in management of RA, nor recommended by RAMRIS so far [11]. As mentioned above, the MRI examination regimen in this study enable bilateral PIPJs scanned more convenient than before. The common MRI findings in PIPJs were synovitis (87% of patients) and tenosynovitis (69% of patients). Flexor pollicis longus tendon extends from the 3rd wrist flexor compartment to IPJ of thumb, while Flexor digitorum profundus and superficialis tendons extend from the 2nd wrist flexor compartment to PIPJ5. MRI tenosynovitis prevalence in IPJ of thumb or PIPJ5 was much higher than the continued wrist flexor compartments, implying tenosynovitis may be more prevalent in PIPJs than wrists. IPJs of thumb or PIPJ2 with MRI synovitis plus tenosynovitis had significantly higher prevalence of tenderness than the corresponding joints with synovitis alone. GEE with multivariate logistic regression confirmed that not only MRI synovitis but also tenosynovitis independently increased more than twice probability of joint tenderness. Since high prevalence of MRI tenosynovitis in PIPJs and its significant contribution to joint tenderness, it demands additional physical examination on the palm of digital joints where tenosynovitis locates, besides for routine examination on the bilateral margin and dorsum. Additionally, more than one third of MRI inflammation in PIPJs was undetectable on physical examination, which demanded PIPJs should be contained in MRI assessment of RA.

There are two main limitations in this study. One is the bias of patient’s enrollment. Since MRI examination was more easily accepted by hospitalized in-patients in China, most of early RA patients enrolled in this study were hospitalized and 51% of them were in high disease activity of RA. Secondly, this is a cross-sectional study with MRI examination in one time-point, and therefore, the outcome of MRI findings could not be evaluated. Further prospective cohort studies are needed to explore the association between dynamic change of MRI examination on bilateral hands and disease progression in early RA patients.

## Conclusions

Given MRI features including vital findings (e.g., abnormality in nervus medianus) are not always symmetric, and overuse may influence certain tenosynovitis in dominant hands, MRI examination of bilateral hands is deserved for disease assessment in early RA patients. The other importance of this study is the detailed description on MRI findings in PIPJs of early RA patients. The higher prevalence of MRI tenosynovitis in PIPJs than the continued wrist flexor compartments and its significant contribution to joint tenderness, demanding additional physical examination on the palm of digital joints and MRI examination regimen, should include PIPJs in management of RA. Finally, we put forward a feasible regimen of MRI examination, which may facilitate bilateral hands including PIPJs into practice in the future.

## Supplementary information

**Additional file 1 : Table S1** The correlation between RAMRIS of bilateral hands and indexes of disease activity or radiographic assessment in 75 early RA patients

## Data Availability

The datasets used and/or analyzed during the current study are available from the corresponding author on reasonable request.

## Abbreviations

RA: Rheumatoid arthritis
MCPJs: Metacarpophalangeal joints
PIPJs: Proximal interphalangeal joints
MRI: Magnetic resonance imaging
FOV: One field of view
RAMRIS: Rheumatoid arthritis magnetic resonance imaging score
ACR: American College of Rheumatology
EULAR: European League Against Rheumatism
DAS28-CRP: 28-joint counts with C-reactive protein
28TJC: 28-joint tender joint count
28SJC: 28-joint swollen joint count
PtGA: Patient global assessment of disease activity
PrGA: Provider global assessment of disease activity
CR: C-reactive protein
ESR: Erythrocyte sedimentation rate
R: Rheumatoid factor
ACPA: Anti-cyclic citrullinated peptide antibody
CDAI: Clinical disease activity index
SDA: Simplified disease activity index
HAQ-DI: Health Assessment Questionnaire-Disability Index
mTSS: Modified total Sharp score
JSN: Joint space narrowing
TR: Time of repetition
TE: Time of echo
IQR: Interquartile range
SD: Standard deviation
GEE: Generalized estimating equations
IPJ: Interphalangeal joint
