# Supplementary material for: The value of MRI examination on bilateral hands including proximal interphalangeal joints for disease assessment in patients with early rheumatoid arthritis: a cross-sectional cohort study
Source: Arthritis Res Ther. 2019 Dec 11;21:279. doi: 10.1186/s13075-019-2061-1 (PMC6907274; doi:10.1186/s13075-019-2061-1)
Supplement: Supplementary file 1 — Additional file 1 : Table S1 The correlation between RAMRIS of bilateral hands and indexes of disease activity or radiographic assessment in 75 early RA patients [file 13075_2019_2061_MOESM1_ESM.docx]

**Table S1 The correlation between RAMRIS of bilateral hands and indexes of disease activity or radiographic assessment in 75 early RA patients**

|  | **28TJC** | **28SJC** | **ESR** | **CRP** | **DAS28-CRP** | **SDAI** | **CDAI** | **Erosion**  **subscore** | **JSN**  **subscore** | **mTSS** |
| --- | --- | --- | --- | --- | --- | --- | --- | --- | --- | --- |
| **RAMRIS** |  |  |  |  |  |  |  |  |  |  |
| **Tenosynovitis** | **0.270*** | 0.190 | 0.141 | **0.269*** | **0.282*** | **0.274*** | 0.209 | 0.141 | 0.038 | 0.116 |
| **Synovitis** | **0.262*** | **0.310**** | **0.339**** | **0.400***** | **0.360**** | **0.339**** | **0.284*** | 0.214 | 0.243 | 0.238 |
| **Osteitis** | 0.14 | 0.079 | 0.179 | **0.242*** | 0.180 | 0.104 | 0.101 | **0.464***** | **0.488***** | **0.522***** |
| **Bone erosion** | **0.349**** | 0.205 | **0.270*** | **0.318**** | **0.390**** | **0.351**** | **0.351**** | **0.416**** | **0.445**** | **0.448**** |

RAMRIS: Rheumatoid arthritis magnetic resonance imaging score; 28TJC: 28-joint tender joint count; 28SJC: 28-joint swollen joint count; ESR: Erythrocyte sedimentation rate; CRP: C-reactive protein; DAS28: Disease activity score in 28 joints; SDAI: Simplified disease activity index; CDAI: Clinical disease activity index; JSN: Joint space narrowing; mTSS: Modified total Sharp score

Spearman rank correlation, **p*<0.05; ***p*<0.01; ****p*<0.001
